# Obesity indices and inflammatory markers in obese non-diabetic normo- and hypertensive patients: a comparative pilot study

**DOI:** 10.1186/1476-511X-13-29

**Published:** 2014-02-08

**Authors:** Mariusz Stępień, Anna Stępień, Rafał N Wlazeł, Marek Paradowski, Maciej Banach, Jacek Rysz

**Affiliations:** 1 Department of Nephrology, Hypertension and Family Medicine, Medical University of Lodz, Żeromskiego str. 113, 90-549 Lodz, Poland; 2 Department of Laboratory Diagnostics and Clinical Biochemistry, Medical University of Lodz, Lodz, Poland; 3 Department of Hypertension, Medical University of Lodz, Lodz, Poland

**Keywords:** Obesity, Hypertension, Chronic inflammation, Obesity indices

## Abstract

**Background:**

The aim of this study was to estimate associations between inflammatory markers and obesity indices in normo- and hypertensive subjects.

**Methods:**

65 obese adult subjects were divided into two groups: (A) of hypertensives (n = 54) and (B) of normotensives (n = 11). Waist circumference (WC), body mass index (BMI), waist-to-hip ratio (WHR), waist-to-height ratio (WHtR), visceral adiposity index (VAI), body adiposity index (BAI) and tumor necrosis factor-α (TNF-α), interleukin (IL)-6 and high sensitivity C-reactive protein (hsCRP) serum concentrations were estimated.

**Results:**

In group A WHtR was higher (0.69 ± 0.07 *vs* 0.63 ± 0.06; *p* < 0.01), hsCRP correlated with BMI and WHtR (r = 0.343; *p* = 0.011 and r = 0.363; *p* < 0.01, respectively). BAI correlated with hsCRP in group A and B (r = 0.329; *p* < 0.05 and r = 0.642; *p* < 0.05; respectively) and in females and males (r = 0.305; *p* = 0.05 and r = 0.44; *p* < 0.05, respectively). In females hsCRP was higher (3.2 ± 2.2 mg/l *vs* 2.1 ± 1.5 mg/l; *p* < 0.05). In patients without lipid lowering treatment hsCRP and IL-6 were higher (3.2 ± 1.7 mg/l *vs* 2.4 ±2.2 mg/l; *p* = 0.01 and 15.9 ± 7.2 pg/ml *vs* 13.6 ± 9.9 pg/ml; *p* < 0.01, respectively).

**Conclusions:**

WHtR is a sensitive index associated with chronic inflammation in obese hypertensive subjects. BAI correlates with hsCRP independently of hypertension and sex. hsCRP is more sensitive marker associated with obesity than IL-6 and TNF-α. Lipid lowering treatment influence chronic inflammation.

## Background

Obesity is one of the most important social problems of communities in developed countries. It has been reported that obesity is associated with a low-grade inflammatory process in the white adipose tissue (WAT) [1-10]. It is a result of chronic activation of the immune system and may contribute to the development of insulin resistance, impaired glucose tolerance or diabetes [1,2]. Increased mass of adipose tissue simultaneously activates the inflammatory process in WAT itself, in the liver and in immune cells [3]. This process leads to increase of circulating levels of proinflammatory cytokines, hormone-like molecules and other inflammatory markers [3]. On the other hand, some mechanisms related mainly to the hypothalamic-pituitary-adrenal axis and autonomic nervous system are activated to counteract this obesity-related stress [3]. Activation of these mechanisms increases levels of glucocorticoids, which may induce the development and differentiation of preadipocytes with further increase of WAT as a result [3]. Moreover, enhanced secretion of proinflammatory cytokines from enlarged WAT may additionally stimulate the hypothalamic-pituitary-adrenal axis [3].

WAT is a source of some proinflammatory cytokines such as tumor necrosis factor alpha (TNF-α) and interleukin-6 (IL-6), which may show both local and systemic effects [1,5-8]. Results of some previously conducted studies indicate that obese WAT is infiltrated by macrophages, which may locally produce inflammatory cytokines [1,2,6,7,10-13]. Macrophage infiltration of adipose tissue increases proportionally to body mass index (BMI), body fat mass and adipocyte hypertrophy, and this effect is reversible on weight loss [7]. Obesity-related chronic inflammation is caused not only by infiltration by inflammatory immune cells but also by a parallel loss or functional reprogramming of immunoregulatory cells [8,10,13]. In normal weighted subjects WAT contains mainly M2 type macrophages which produce anti-inflammatory cytokines such as IL-10, IL-1 Ra and arginase (inhibiting enzyme of nitric oxide synthase – iNOS), whereas in obesity-related WAT, especially of visceral origin, M1 type macrophages dominate [3,8,10,11]. This type of macrophage is characterized by a specific surface marker (CD11c+) and by production of iNOS and classical proinflammatory markers [3]. Type M1 macrophages are stimulated by interferon-γ and lipopolysaccharides to release TNF-α and IL-6 and to produce reactive oxygen species (ROS) [11]. Thus, obesity-related inflammation is associated with a switch from the M2 to M1 phenotype, especially in visceral adipose tissue, which forms “crown-like structures” (CLS) constituted by dead adipocytes and adipocyte cellular fragments [2,3,12].

Rapid increase of fat mass in obese subjects may be associated with hypoperfusion and adipose hypoxia which may induce both oxidative and endoplasmic reticulum stress contributing to release of inflammatory mediators [3,7,9]. Enhanced expression of proinflammatory cytokines may also be a result of increased activation of both c-jun N-terminal kinase and inhibitor of k-kinase [4]. Obesity-related changes were observed in inflammasome, which is a macromolecular innate immune cell sensor initiating the inflammatory response [4]. One of the structural parts of inflammasome are monomers containing NOD-like receptors (NLRs) and one of them, NLRP3, is related to metabolic stress, insulin resistance and type 2 diabetes [4]. Activation of NLRP3 leads to stimulation of macrophage-mediated T cells in adipose tissue with impairment of insulin sensitivity as a result [4]. Hyperglycemia and some substances such as reactive oxygen species (ROS), palmitate, lipopolysaccharides and uric acid can induce inflammasome activation [4].

TNF-α, IL-6 and the inflammasome-activated IL-1β are the most important cytokines responsible for the chronic inflammatory process [4-6,12,13]. Elevated levels of circulating TNF-α were observed in obese subjects [4-14]. TNF-α is a cytokine not only associated with inflammation but also plays an important role in the development of the immune system, induces adipocyte apoptosis and also shows numerous effects in adipose tissue, especially in lipid metabolism and insulin signaling [4,6,8,10,13,14]. TNF-α stimulates secretion of another proinflammatory cytokine, IL-6 [4,10-14]. IL-6 is produced by adipose tissue, endothelial cells (especially by the stromal vascular fraction), fibroblasts, macrophages, monocytes and lymphocytes, and contributes to acute phase reactions and chronic inflammatory processes, regulation of energy homeostasis and to other important processes [4,6,10,13,14]. This cytokine also plays an important role in chronic inflammation in both obesity and insulin resistance [4,6,10,13,14].

C-reactive protein (CRP) is a very sensitive marker of inflammation, which is synthesized in the liver, and this process is regulated predominately by IL-6 [4,6]. CRP is positively correlated with abdominal fat and closely correlated with increased risk of cardiovascular events [4,12].

Results obtained from some studies indicate that adipose body mass is related to chronic inflammation and that visceral obesity is associated with elevated CRP levels measured by a high-sensitivity method (hsCRP) independently from BMI [4,15]. Circulating levels of CRP, IL-6 and TNF-α were elevated in healthy obese subjects in adults and also in children compared to normal weighted volunteers [4,13].

The causes and mechanisms of obesity-induced chronic inflammation as well as the association between inflammatory markers or cytokines and obesity parameters are still not fully explained. It is worth noting that obesity very often coexists with hypertension, which is also associated with a chronic inflammatory process [15-19]. Therefore the aim of this study was to evaluate associations between inflammatory markers or cytokines such as IL-6, TNF-α and hsCRP, and both classical and newer obesity parameters and indices in obese normo- and hypertensive subjects.

## Material and methods

Sixty-five obese (BMI ≥ 30 kg/m^2^) non-diabetic Caucasian out-patients of both sexes (25 males and 40 females) aged from 35 to 64 years were enrolled in the study. Patients were consecutively recruited between the 1^st^ of June and 15^th^ of December 2010. Patients with acute or chronic inflammation or those with hsCRP levels above 10 mg/l, psychiatric disorders, pregnancy, cancer, severe hepatic or renal diseases and acute cardiovascular events or with a history of abdominal surgery, which could have any impact on abdominal fat distribution, were excluded from the study.

Participants were divided into 2 basic groups: (A) – hypertensives (n = 54); and (B) – normotensives (n = 11). All the subjects were divided according to serum levels of hsCRP into the following 3 groups: group 1 – patients with low cardiovascular risk (CVR) (hsCRP < 1 mg/l), n = 11; group 2 – patients with medium CVR (hsCRP between 1 and 3 mg/l), n = 30; and group 3 with high CVR (hsCRP > 3 mg/l), n = 24. All the patients were additionally analysed on the basis of sex: males (n = 20) and females (n = 35), to estimate sex-dependent differences. All the study participants were also divided into 2 groups: those receiving hypolipidemic agents (statins, fibrates or both) and not receiving lipid lowering treatment to estimate the impact of this therapy on serum levels of hsCRP, IL-6 and TNF-α.

After taking their medical history all the subjects had blood pressure (BP) and pulse measured 3 times after a 10 min rest with an OMRON M4 Plus automated device using the left upper arm and averaged. All the study participants were evaluated for their body mass (BM), height, waist (measured at a level midway between the lowest rib and the liac crest) and hip (widest diameter over greater trochanters) circumferences (WC and HC, respectively) to calculate obesity indices as follows:

1) ***Body mass index***

BMI = weight/height^2^ (kg/m^2^)

2) ***Waist-to-hip ratio***

WHR = waist circumference (cm)/hip circumference (cm)

3) ***Waist-to-height ratio***

WHtR = waist (cm)/height (cm)

Normally ranges from 0.46 to 0.62 [20]

4) ***Visceral adiposity index***

VAI for:

$$\mathrm{Males}=\left(\frac{\mathrm{WC}}{39.68+\left(1.88\times \mathrm{BMI}\right)}\right)\times \left(\frac{\mathrm{TGs}}{1.03}\right)\times \left(\frac{1.31}{\mathrm{HDL}‒C}\right)$$

$$\mathrm{Females}=\left(\frac{\mathrm{WC}}{36.58+\left(1.89\times \mathrm{BMI}\right)}\right)\times \left(\frac{\mathrm{TGs}}{0.81}\right)\times \left(\frac{1.52}{\mathrm{HDL}‒C}\right)$$

Normal value = 1 in healthy non-obese subjects with normal adipose distribution and normal TG and HDL-C levels [21];

5) ***Body adiposity index***

$$\mathrm{BAI}=\frac{\mathrm{Hip}\;\mathrm{circumference}}{\mathrm{Height}{\left(m\right)}^{1.5}}-18$$

in which hip circumference is measured in cm [22].

Serum TNF-α and IL-6 concentrations were estimated using an ELISA method (DRG Instruments GmbH, Marburg, Germany) in fasting venous blood samples. Serum high-sensitivity C-reactive protein (hsCRP) levels were measured by immunoturbidimetry. Serum levels of triglyceride (TG) and high-density lipoprotein cholesterol (HDL-C) were measured to calculate VAI.

The study protocol was approved by the local Bioethical Committee and informed consent was obtained from all the patients.

### Statistical analysis

Results are presented as means ± standard deviation (SD). Some of the data were also expressed as proportions (%). The assumption of normality was assessed with the Shapiro-Wilk test. Homogeneity of variances was verified with Levene’s test. Significance of differences between means from two compared groups were determined by Student’s t-test or by one-way analysis of variances (ANOVA) with post-hoc Scheffé test for detailed multiple comparisons (more than two groups). Chi-square tests were used for comparison of categorical variables. When the distribution of the variables was not normal in at least one of the compared groups Mann-Whitney U-test and Kruskal-Wallis analysis of variances with post-hoc Conover-Inman tests were applied. Spearman's rank correlation coefficient (r) was used to estimate the correlation between variables from comparative groups. P values <0.05 were considered significant.

## Results

Clinical characteristics of investigated groups are presented in Tables 1, 2 and 3 and the results are presented in Tables 4, 5, 6, 7, 8, 9, 10 and 11.

**Table 1:** Clinical characteristics of obese hypertensive and normotensive patients

**Compared groups and clinical data**	**Group A**	**Group B**	**Statistical significance p**
	**n = 54**	**n = 11**	
	**n (%)**	**(%)**	
**Males/females**	20 (37.0%)/34 (63.0%)	5 (45.5%)/6 (54.5)	NS
**Age (years)**	57.9 ± 11.0	46.1 ± 10.8	p = 0.001
**Ischemic heart disease**	18 (33.3%)	2 (18.2%)	NS
**Myocardial infarct**	7 (13.0%)	1 (9.1%)	NS
**Cardiac failure**	22 (40.7%)	3 (27.3%)	NS
**IFG/IGT**	34 (63.0%)	4 (36.4%)	NS
**CKD**	14 (25.9%)	1 (9.1%)	NS
**Stroke**	3 (5.6%)	0	NS
**Cerebral arteries failure**	3 (5.6%)	0	NS
**Hyperuricemia**	24 (44.4%)	2 (18.2%)	NS
**Dyslipidaemia**	52 (96.3%)	9 (81.8%)	NS
**Tobacco smoking**	5 (9.3%)	0	NS
**ACEI**	44 (81.5%)	1 (9.1%)	p < 0.0001
**ARB**	17 (31.5%)	0	p = 0.05
**Diuretics**	35 (64.8%)	1 (9.1%)	p < 0.01
**CCA**	19 (35.2%)	0	p < 0.05
**BB**	23 (40.7%)	2 (18.2%)	NS
**Spironolactone**	17 (31.5%)	1 (9.1%)	NS
**ARA**	7 (13.0%)	0	NS
**ASA**	15 (27.8%)	1 (9.1%)	NS
**Hypolipemic treatment**^**a**^	31 (57.4%)	4 (36.4%)	NS

IFG – impaired fasting glucose. IGT – impaired glucose tolerance. CKD – chronic kidney disease. COPD – chronic pulmonary obstructive disease. ACEI – angiotensin-converting enzyme inhibitor. ARB – angiotensin II receptor antagonist. CCA – calcium channel antagonist. BB – β-adrenergic receptor blocker. ARA – α_1_-adrenergic receptor antagonist. ASA – acetylsalicylic acid. NS – not significant. ^a^ – statins and/or fibrates.

**Table 2:** Clinical characteristics of the patient groups according to hs-CRP level (cardiovascular risk)

**Compared groups and clinical data**	**Group 1**	**Group 2**	**Group 3**
	**low CVR**	**medium CVR**	**high CVR**
	**n = 11**	**n = 30**	**n = 24**
**Males**	7 (63.6%)	14 (46.7%)	4 (16.7%)
**Females**	4 (36.4%)	16 (53.3%)	20 (83.3%)*^
**Ages (years)**	56.6 ± 12.7	55.4 ± 9.9	57.2 ± 13.0
**Hypertension**	8 (72.7%)	26 (86.7%)	20 (83.3%)
**Ischemic heart disease**	3 (27.3%)	11 (36.7%)	6 (25.0%)
**Myocardial infarct**	1 (9.1%)	6 (20.0%)	1 (4.2%)
**Cardiac failure**	3 (27.3%)	11 (36.7%)	11 (45.8%)
**IFG/IGT**	5 (45.5%)	21 (70.0%)	12 (50.0%)
**CKD**	1 (9.1%)	7 (23.3%)	7 (29.2%)
**Stroke**	0	1 (3.3%)	2 (8.3%)
**Cerebral arteries failure**	1 (9.1%)	2 (6.7%)	0
**Hyperuricemia**	3 (27.3%)	11 (36.7%)	12 (50.0%)
**Dyslipidemia**	11 (100.0%)	29 (96.7%)	21 (87.5%)
**Tobacco smoking**	0	3 (10.0%)	2 (8.3%)
**ACEI**	9 (81.8%)	27 (90.0%)	11 (45.8%)^^
**ARB**	1 (9.1%)	6 (20.0%)	10 (41.7%)
**Diuretics**	6 (54.5%)	16 (53.3%)	16 (66.7%)
**CCA**	4 (36.4%)	10 (33.3%)	6 (25.0%)
**BB**	3 (27.3%)	10 (33.3%)	12 (50.0%)
**Spironolactone**	2 (18.2%)	5 (16.7%)	11 (45.8%)^
**ARA**	2 (18.2%)	4 (13.3%)	1 (4.2%)
**ASA**	1 (9.1%)	11 (36.7%)	5 (20.8%)
**Hypolipemic treatment**^**a**^	10 (90.9%)	*15 (50.0%)	**10 (41.7%)

*p < 0.05; **p < 0.01; vs Group 1; ^P < 0.05 vs Group 2; ^^P < 0.01 vs Group 2; CVR – cardiovascular risk, ^a^ – statins and/or fibrates.

**Table 3:** Clinical characteristics of investigated group divided by sex

**Compared groups and clinical data**	**Females**	**Males**	**Statistical significance p**
	**n = 40**	**n = 25**	
**Age (years)**	57.9 ± 11.0	52.6 ± 12.3	NS
**Hypertension**	34 (85.0%)	20 (80.0%)	NS
**Ischemic heart disease**	9 (22.5%)	11 (44.0%)	NS
**Myocardial infarct**	2 (5.0%)	6 (24.0%)	p < 0.05
**Cardiac failure**	18 (45.0%)	7 (28.0%)	p < 0.05
**IFG/IGT**	21 (52.5%)	17 (68.0%)	NS
**CKD**	13 (32.5%)	2 (8.0%)	p < 0.05
**Stroke**	2 (5.0%)	1 (4.0%)	NS
**Cerebral arteries failure**	0	3 (12.0%)	p = 0.053
**Hyperuricemia**	17 (42.5%)	14 (56.0%)	NS
**Dyslipidemia**	37 (92.5%)	24 (96.0%)	NS
**Tobacco smoking**	2 (5.0%)	3 (12.0%)	NS
**ACEI**	31 (77.5%)	15 (60.0%)	NS
**ARB**	13 (32.5%)	4 (16.0%)	NS
**Diuretics**	27 (67.5%)	10 (40.0%)	p < 0.05
**CCA**	12 (30.0%)	7 (28.0%)	NS
**BB**	17 (42.5%)	8 (32.0%)	NS
**Spironolactone**	14 (35.0%)	4 (16.0%)	NS
**ARA**	1 (2.5%)	6 (24.0%)	p = 0.01
**ASA**	10 (25.0%)	7 (28.0%)	NS
**Hypolipemic treatment**^**a**^	20 (50.0%)	15 (60.0%)	NS

^a^ – statins and/or fibrates.

**Table 4:** Results obtained in obese hypertensive and normotensive patients

**Parameters**	**Group A**	**Group B**	**Statistical significance p**
	**N = 54**	**N = 11**	
**SBP (mmHg)**	139.2 ± 16.5	121.3 ± 9.9	p = 0.001
**DBP (mmHg)**	86.7 ± 9.5	81.1 ± 5.2	NS
**HR (min**^**-1**^**)**	74.7 ± 10.1	77.8 ± 8.1	NS
**WC (cm)**	112.8 ± 11.5	106.6 ± 8.7	NS
**WHR**	0.95 ± 0.08	0.92 ± 0.05	NS
**WHTR**	0.69 ± 0.07	0.63 ± 0.06	p < 0.01
**BMI (kg/m**^**2**^**)**	36.5 ± 4.8	34.7 ± 3.1	NS
**BAI**	39.3 ± 7.4	35.0 ± 6.1	NS
**VAI**	2.1 ± 1.1	1.9 ± 1.0	NS
**hs-CRP (mg/l)**	2.8 ± 1.9	2.7 ± 2.5	NS
**IL-6 (pg/ml)**	14.6 ± 9.1	14.9 ± 7.3	NS
**TNF-α (pg/ml)**	6.2 ± 1.8	6.7 ± 1.9	NS

SBP – systolic blood pressure, DBP – diastolic blood pressure.

**Table 5:** Correlations between estimated parameters in group A (n = 54)

**Parameters**	**hs-CRP**	**IL-6**	**TNF-α**
	**(mg/l)**	**(pg/ml)**	**(pg/ml)**
**WC (cm)**	0.211	-0.021	0.111
	NS	NS	NS
**BMI (kg/m**^**2**^**)**	0.343	0.054	0.185
	p = 0.011	NS	NS
**WHR**	-0.107	-0.075	-0.093
	NS	NS	NS
**WHTR**	0.363	0.070	0.187
	p < 0.01	NS	NS
**BAI**	0.329	0.098	0.094
	p < 0.05	NS	NS
**VAI**	0.188	0.281	0.229
	NS	p < 0.05	NS

Data are expressed as r – Spearman’s rank correlation with statistical significance p.

**Table 6:** Correlations between estimated parameters in group B (n = 11)

**Parameters**	**hs-CRP**	**IL-6**	**TNF-α**
	**(mg/l)**	**(pg/ml)**	**(pg/ml)**
**WC (cm)**	-0.297	0.069	0.254
	NS	NS	NS
**BMI (kg/m**^**2**^**)**	-0.014	0.046	0.292
	NS	NS	NS
**WHR**	-0.429	0.329	0.126
	NS	NS	NS
**WHTR**	0.393	0.600	-0.261
	NS	P = 0.05	NS
**BAI**	0.642	0.347	-0.265
	p < 0.05	NS	NS
**VAI**	0.333	0.455	-0.167
	NS	NS	NS

Data are expressed as r – Spearman’s rank correlation with statistical significance p.

**Table 7:** Results obtained in the patient groups divided according to hs-CRP level (cardiovascular risk)

**Compared data**	**Group 1**	**Group 2**	**Group 3**
	**(low CVR)**	**(medium CVR)**	**(high CVR)**
	**n = 11**	**n = 30**	**n = 24**
**RRs (mmHg)**	135.0 ± 18.3	132.7 ± 14.1	141.0 ± 19.1
**RRr (mmHg)**	84.1 ± 10.6	84.2 ± 7.8	88.6 ± 9.7
**HR (min**^**-1**^**)**	75.3 ± 9.1	75.4 ± 10.7	75.0 ± 9.4
**WC (cm)**	109.3 ± 9.7	111.5 ± 11.1	113.2 ± 12.3
**WHR**	0.98 ± 0.10	0.95 ± 0.08	0.92 ± 0.06
**WHTR**	0.64 ± 0.06	0.67 ± 0.06	**0.71 ± 0.07#
**BMI (kg/m**^**2**^**)**	33.4 ± 1.8	35.7 ± 4.0	*38.1 ± 5.4
**BAI**	33.3 ± 5.9	*37.1 ± 5.3	**42.8 ± 8.1##
**VAI**	1.7 ± 1.0	2.2 ± 1.2	2.1 ± 1.0
**hsCRP (mg/l)**	0.83 ± 0.26	***1.8 ± 0.6	***4.9 ± 1.7###
**IL6 (pg/ml)**	10.0 ± 3.5	13.9 ± 8.9	**17.7 ± 9.4#
**TNF-α (pg/ml)**	6.7 ± 2.8	6.3 ± 1.6	6.1 ± 1.4

*p < 0.05; **p < 0.01; ***P < 0.0001 vs Group 1; #p < 0.05; ##p < 0.01; ###P < 0.0001 vs Group 2.

**Table 8:** Results obtained in the patient groups divided by sex

**Compared data**	**Females**	**Males**	**Statistical significance p**
	**n = 40**	**n = 25**	
**Hypolipidemic treatment**	20 (50.0%)	15 (60.0%)	NS
**SBP (mmHg)**	137.1 ± 17.7	134.5 ± 15.9	NS
**DBP (mmHg)**	86.0 ± 9.4	85.5 ± 8.9	NS
**HR (min**^**-1**^**)**	75.1 ± 9.6	75.5 ± 10.3	NS
**WC (cm)**	108.5 ± 10.4	117.0 ± 10.8	p < 0.01
**WHR**	0.91 ± 0.06	1.01 ± 0.06	P < 0.0001
**WHTR**	0.68 ± 0.07	0.67 ± 0.06	NS
**BMI (kg/m**^**2**^**)**	36.2 ± 4.8	36.1 ± 4.3	NS
**BAI**	41.7 ± 6.9	33.5 ± 5.0	P < 0.0001
**VAI**	2.0 ± 1.0	2.1 ± 1.3	NS
**hs-CRP (mg/l)**	3.2 ± 2.2	2.1 ± 1.5	p < 0.05
**IL-6 (pg/ml)**	16.7 ± 10.4	11.5 ± 3.7	NS
**TNF-α (pg/ml)**	6.2 ± 1.4	6.5 ± 2.2	NS

**Table 9:** Correlations between estimated parameters in female groups (n = 40)

**Parameters**	**hs-CRP**	**IL-6**	**TNF-α**
	**(mg/l)**	**(pg/ml)**	**(pg/ml)**
**WC (cm)**	0.265	0.094	0.269
	NS	NS	NS
**BMI (kg/m**^**2**^**)**	0.305	0.075	0.251
	P = 0.05	NS	NS
**WHR**	0.038	0.171	0.023
	NS	NS	NS
**WHTR**	0.321	0.071	0.294
	p < 0.05	NS	NS
**BAI**	0.309	-0.009	0.211
	P = 0.05	NS	NS
**VAI**	0.128	0.239	0.143
	NS	NS	NS

Data are expressed as r – Spearman’s rank correlation with statistical significance p.

**Table 10:** Correlations between estimated parameters in male groups (n = 25)

**Parameters**	**hs-CRP**	**IL-6**	**TNF-α**
	**(mg/l)**	**(pg/ml)**	**(pg/ml)**
**WC (cm)**	0.362	0.023	-0.074
	NS	NS	NS
**BMI (kg/m**^**2**^**)**	0.367	-0.061	0.014
	NS	NS	NS
**WHR**	0.262	0.062	-0.408
	NS	NS	p < 0.05
**WHTR**	0.458	0.066	-0.191
	p < 0.05	NS	NS
**BAI**	0.440	0.071	-0.135
	p < 0.05	NS	NS
**VAI**	0.443	0.472	0.142
	p < 0.05	p < 0.05	NS

Data are expressed as r – Spearman’s rank correlation with statistical significance p.

**Table 11:** Results obtained in the patient groups divided by receiving hypolipemic treatment

**Parameters**	**Hypolipemic treatment**	**Without hypolipemic treatment**	**Statistical significance p**
	**(N = 35)**	**(N = 30)**	
**Hypertension**	31 (88.6%)	23 (76.7%)	NS
**Age (years)**	58.8 ± 11.1	52.4 ± 11.7	p < 0.01
	61.0 (56.0-64.0)	54.0 (45.5-60.8)	
**Males**	15 (42.9%)	10 (33.3%)	NS
**hs-CRP (mg/l)**	2.4 ± 2.2	3.2 ± 1.7	P = 0.010
	1.7 (1.0-3.5)	3.0 (1.8-4.4)	
**IL-6 (pg/ml)**	13.6 ± 9.9	15.9 ± 7.2	p < 0.01
	10.3 (8.9-14.5)	14.5 (10.3-18.9)	
**TNF-α (pg/ml)**	6.3 ± 1.7	6.3 ± 1.9	NS
	6.3 (5.5-7.1)	6.0 (4.9-7.1)	

Hypertensive obese patients (group A) were older than normotensive participants from group B (57.9 ± 11.0 *vs* 46.1 ± 10.8 years; *p* = 0.001; Table 1) and in comparison to group B they more often received angiotensin-converting enzyme inhibitors (ACEI) (81.5% *vs* 9.1%; *p* < 0.0001), angiotensin II receptor antagonists (ARB) (*p* = 0.05), diuretics (64.8% *vs* 9.1%; *p* < 0.01) and calcium channel antagonists (CCA) (*p* < 0.05) (Table 1).

Females dominate in group 3 with high cardiovascular risk (CVR) compared to group 1 and 2 (83.3% *vs* 36.4%; *p* < 0.05 and 83.3% *vs* 53.3%; *p* < 0.05, respectively; Table 2). Subjects from group 3 compared to group 2 received more seldom ACEI but more often spironolactone (45.8% *vs* 90.0%; *p* < 0.01 and 45.8% *vs* 16.7%; *p* < 0.05, respectively; Table 2). Subjects with low CVR from group 1 received more often hypolipemic drugs (statins, fibrates) compared to group 2 and 3 (90.9% *vs* 50.0%; *p* < 0.05 and 90.9% *vs* 41.7%; *p* < 0.01, respectively; Table 2).

Myocardial infarction and cerebral artery failure occurred more often in obese males compared to females (24.0% *vs* 5.0%; *p* < 0.05 and 12.0% *vs* 0%; *p =* 0.053, respectively; Table 3). On the other hand, obese females suffered more often from cardiac failure and chronic kidneys disease (CKD) (45.0% *vs* 28.0%; *p* < 0.05 and 32.5% *vs* 8,0%; *p* < 0.05, respectively; Table 3). Females received more often diuretics but more rarely α_1_-adrenergic receptor antagonists than males (67.5% *vs* 40.0%; *p* < 0.05 and 2.5% *vs* 24.0%; p = 0.01, respectively; Table 3).

Obese hypertensive patients (group A) had higher mean systolic blood pressure (SBP) compared to normotensive subjects from group B (139.2 ± 16.5 mmHg *vs* 121.3 ± 9.9 mmHg; *p* = 0.001; Table 4). Mean WHtR was higher in group A compared to group B (0.69 ± 0.07 *vs* 0.63 ± 0.06; *p* < 0.01; Table 4). In group A hsCRP serum levels positively correlated with BMI, WHtR and BAI (r = 0.343; *p* = 0.011; r = 0.363; *p* < 0.01 and r = 0.329; *p* < 0.05, respectively) and IL-6 serum concentrations positively correlated with VAI (r = 0.281; *p* < 0.05) (Table 5).

In group B positive correlations were observed between hsCRP serum levels and BAI as well as between IL-6 serum concentrations and WHtR (r = 0.642; *p* < 0.05 and r = 0.6; *p* = 0.05, respectively; Table 6).

Mean WHtR was higher in patients with high CVR from group 3 compared to group 1 and 2 (0.71 ± 0.07 *vs* 0.64 ± 0.06; *p* < 0.01 and 0.71 ± 0.07 *vs* 0.67 ± 0.06; *p* < 0.05, respectively; Table 7). Mean BMI was higher in group 3 compared to group 1 (38.1 ± 5.4 kg/m^2^*vs* 33.4 ± 1.8 kg/m^2^; *p* < 0.05; Table 7). Mean BAI was higher in group 3 than in group 1 and 2 (42.8 ± 8.1 *vs* 33.3 ± 5.9; *p* < 0.01 and 42.8 ± 8.1 *vs* 37.1 ± 5.3; *p* < 0.01, respectively; Table 7). Mean hsCRP serum concentration was higher in group 3 in comparison to groups 1 and 2 (4.9 ± 1.7 mg/l *vs* 0.83 ± 0.26 mg/l; *p* < 0.0001 and 4.9 ± 1.7 mg/l *vs* 1.8 ± 0.6 mg/l; *p* < 0.0001, respectively; Table 7). Mean hsCRP serum level was higher in group 2 compared to group 1 (1.8 ± 0.6 mg/l; *p* < 0.0001; Table 7). Mean IL-6 serum concentration was higher in group 3 than in group 1 and 2 (17.7 ± 9.4 pg/ml *vs* 10.0 ± 3.5 pg/ml; *p* < 0.01 and 17.7 ± 9.4 pg/ml *vs* 13.9 ± 8.9 pg/ml; *p* < 0.05, respectively; Table 7).

In males mean WC and mean WHR were higher than in females (117.0 ± 10.8 cm *vs* 108.5 ± 10.4 cm; *p* < 0.01 and 1.01 ± 0.06 *vs* 0.91 ± 0.06; *p* < 0.0001, respectively; Table 8). On the other hand, in females mean BAI and hsCRP serum concentrations were higher than in the male group (41.7 ± 6.9 *vs* 33.5 ± 5.0; *p* < 0.0001 and 3.2 ±2.2 mg/l *vs* 2.1 ± 1.5 mg/l; *p* < 0.05, respectively; Table 8).

In females hsCRP serum levels positively correlated with WHtR (r = 0.321; *p* < 0.05) and BMI and BAI (r = 0.305; *p* = 0.05, and r = 0.309; *p* = 0.05, respectively; Table 9). In males hsCRP serum concentrations positively correlated with WHtR, BAI and VAI (r = 0.458; *p* < 0.05; r = 0.440; *p* < 0.05 and r = 0.443; *p* < 0.05, respectively), IL-6 serum concentrations positively correlated with VAI (r = 0.472; *p* < 0.05) and TNF-α serum concentrations negatively correlated with WHR (r = - 0.408; *p* < 0.05) (Table 10).

Patients receiving hypolipemic treatment (statins, fibrates or both) were older compared to the subjects without this treatment (58.8 ± 11.1 years *vs* 52.4 ± 11.7 years; p < 0.01; Table 11). In the group of patients not receiving hypolipemic agents mean serum concentrations of hsCRP and IL-6 were higher compared to the patients receiving hypolipemic treatment (3.2 ± 1.7 mg/l *vs* 2.4 ± 2.2 mg/l; *p* = 0.01 and 15.9 ± 7.2 pg/ml *vs* 13.6 ± 9.9 pg/ml; *p* < 0.01, respectively; Table 11).

## Discussion

It was reported that both hypertension and obesity are associated with chronic inflammation [1-18]. Therefore one could expect significant differences in serum levels of hsCRP, IL-6 and TNF-α between obese normotensive and hypertensive subjects. Nevertheless, we did not observe any significant difference in the serum mean concentrations of inflammatory markers between the compared groups. We suggest that it might result from the small sizes of both groups, especially of normotensive participants. We cannot exclude that it might also be related to angiotensin-converting enzyme inhibitors (ACEI) and angiotensin II receptor antagonists (sartans, ARB), which significantly dominated in the group of hypertensive obese patients. There is more evidence that angiotensin II blockade by both ACEI and ARB may significantly reduce concentrations of proinflammatory mediators and oxidative stress products in numerous models of inflammation; however, the precise mechanism of these effects is not fully explained [16,17]. We can also speculate that hypolipemic treatment [more often in the hypertensive group] might also have an impact on the result; however, the difference observed between the groups did not reach statistical significance.

The results of our study suggest that WHtR, a new index of visceral obesity, may be a more sensitive obesity index associated with chronic inflammation in obese hypertensive patients. WHtR was also higher in obese patients with high and medium CVR. Lapice *et al.* observed a relationship between abdominal obesity (WHR, WC) and hsCRP independently of BMI and sex in non-diabetic subjects with abdominal obesity [23]. Arbel *et al*. in a large population (n = 13,033) study found associations between hsCRP and obesity indices such as WC, BMI, BAI, WHR and WHtR, where the strongest correlations were observed for BMI, WC and WHtR [24]. Significant correlations between hsCRP and BMI, WHtR and BAI in obese subjects were also noted in our previously conducted study [25]. The aforementioned Arbel *et al.* also found higher hsCRP in females compared to males [24]. Similar to these findings we observed higher hsCRP in females and we suppose that it might result from higher adiposity, described by BAI, which was higher in women. Our findings are in agreement with results published by Pannacciulli *et al.,* who observed a positive association between hsCRP and total body fat as well as with central fat in adult women [26]. Therefore we suggest that obesity chronic inflammation is more evident in the women compared to the men.

Strong correlations between hsCRP and BAI as well as with BMI and WC were observed by Lichtash *et al.* in adult Mexican Americans independently of the sex [27]. These results are confirmed by our observations for BMI and BAI in hypertensive obese patients, although we found some sex-dependent differences for BMI (a significant association only in females) and with the correlation between hsCRP and BAI on the border of statistical significance in females. Result obtained in our present study from obese hypertensive subjects as well as from the female group are in agreement with data published by Thung-Wei *et al.*[28]. The authors based on data from the NHANES study [n = 8 453] found a strong positive correlation between BMI and hsCRP in adult participants [28]. Results obtained in our study are also in agreement with data published by Engeli *et al.,* who observed a strong correlation between hsCRP and BMI in obese women [29].

Similarly as in our previously conducted studies we did not observe significant differences in BMI between sexes [19,30]. Nevertheless, parameters of visceral obesity such as WC and WHR are higher in females than in males, whereas BAI – a parameter of adiposity – was higher in women. These results are in agreement with our previously obtained results and with the data reported by other authors, who did find sex-dependent differences in distribution of fat tissue, involving both size and number of adipocytes [19,30,31]. Adipocytes from gluteo-femoral fat tissue in females are more numerous and larger whereas visceral adipocytes are smaller than in males [31]. However, the sizes of abdominal subcutaneous adipocytes are comparable between both sexes [31]. Sex-dependent differences in fat distribution are well established [31,32]. In comparison to males, who often have more fat tissue in the central or abdominal region, females have more body fat with relatively greater distribution in the hips and thighs [31,32]. Compared to the men, women have more subcutaneous adipose tissue and less visceral adipose tissue [32]. Adipose tissue distribution in females changes with age. Therefore in postmenopausal women there is an increased amount of visceral adipose tissue [32].

It should be taken into consideration that although fat accumulation in the subcutaneous abdominal area is associated with adipocyte hypertrophy in both women and men, females primarily started with a greater number of adipocytes which can later accumulate as greater fat mass [31]. It is well known that both human adipocytes and preadipocytes express sex steroid receptors [31,32]. Nevertheless, it was suggested that sex hormones primarily affect adipose tissue indirectly by the central nervous system [31,32]. Results obtained from *in vitro* studies indicate that estrogens stimulate proliferation of human preadipocytes in contrast to androgens, which inhibit differentiation of these cells without affecting proliferation [31].

Based on the data obtained from our present study, we suggest that adiposity, especially described by BAI, may be related to chronic inflammation independently from hypertension and sex. Moreover, WHtR – a newer index of visceral obesity – may be a more sensitive predictor of obesity-related chronic inflammation in both sexes as well as in hypertensive obese patients than classical parameters such as WC and WHR. In obese males VAI may be a valuable new parameter of visceral obesity associated with the chronic inflammatory process.

Park *et al.* observed that CRP was significantly associated with BMI whereas IL-6 better correlated with visceral obesity in obese subjects [33]. These findings partially agree with some results obtained in our study. We observed a significant correlation between IL-6 only with some newer indices of visceral obesity such as VAI and WHtR, whereas hsCRP also significantly correlated with BMI and BAI. We suppose that the lack of significant correlations between classical visceral obesity indices such as WC or WHtR and markers of inflammation observed in our study might result from the small sizes of the groups. We suggest that newer visceral obesity indices such as WHtR and VAI may be more sensitive visceral obesity indices, as proposed by others [20,21]. In contrast to our findings, Saijo *et al.* observed significant correlations between CRP and classical visceral obesity parameters such as WC and WHR [34].

Strong correlations between BMI and both hsCRP and TNF-α serum levels were observed by Khan *et al.* in non-diabetic obese subjects [35]. The authors also suggested that TNF-α may be associated with CVR [35]. Nevertheless, we did not observe significant differences in mean TNF-α levels between patient groups divided according to CVR. Based on results obtained in our study it seems that IL-6 similarly to hsCRP is more associated with CVR than TNF-α. Higher mean CRP plasma level and a positive correlation between IL-6 and WHtR was found by Gharipour *et al.* in subjects with metabolic syndrome; however, the authors did not observe significant relationships between CRP levels and obesity parameters such as BMI, WHtR and WC in this study group [36]. We noted a similar association between IL-6 and WHtR, but on the border of statistical significance in the obese normotensive group. It is worth noting that Gharipour *et al.* did not distinguish subgroups for those with or without hypertension in the population of patients with metabolic syndrome [36].

In our study BMI and WC did not correlate with IL-6 and TNF-α in any investigated subgroups. These findings are in agreement with results obtained by Agraval *et al.,* who also did not observe any significant correlation between IL-6 and TNF-α with BMI and WC in a North Indian healthy general population as well in both sexes; however, the TNF-α serum level was higher in obese subjects compared to non-obese participants [37]. In contrast we observed a significant inverse correlation between WHR and TNF-α in the obese male group. Based on the data obtained from our study it is difficult to explain this surprising relationship.

The results of our study confirm the anti-inflammatory effect of statins and fibrates. Hypolipemic treatment led to significant decrease of hsCRP and IL-6 mean serum levels but without any impact on TNF-α serum concentration. These findings are in agreement with previously published data [38-40]. All the authors cited above observed a similar impact of statins or fibrates on hsCRP and IL-6 without changing plasma or serum levels of TNF-α [38-40]. Hypolipemic treatment might also have an impact on obtained results in the groups divided according hsCRP level. In the group of subjects with high and medium CVR we observed higher BMI, WHtR and BAI as well as higher IL-6 mean serum levels compared to the group with low CVR. These results confirm the relationships between mentioned above obesity parameters and hsCRP in other study groups. It should be taken into consideration that sex and some agent might have an impact on these results. Its worth noting that in group 3 with high CVR females dominated and the subjects from this group received more seldom lipid lowering agents as well as ACEI compared to the other groups. As mentioned above, both groups of these agents may have an impact on chronic inflammation. On the other hand, higher values of BMI, WHtR and BAI may be indicators of CVR, as was previously reported [20-28,33,35].

The mechanism of anti-inflammatory action of lipid lowering agents remains not fully explained. It is well documented that IL-6 is produced by adipocyte tissue in proportion to fat mass and hsCRP plasma levels are also associated with adipose tissue [40]. However, it was not established whether fibrates reduced hsCRP directly by their impact on the liver or on the vascular bed [38]. It has been suggested that fenofibrate may reduce systemic inflammation by actions on multiple tissues [38]. It is proposed that fibrates lead to increase of IL-6 synthesis via PPAR-α receptors with reduction of hsCRP plasma levels as a result [38]. It was reported that PPAR-α receptors are abundant in the vascular bed and fenofibrate may improve endothelial function as well as vascular reactivity by decreasing ICAM/VCAM plasma levels [38]. Some authors suggest that statins may inhibit macrophage activity and subsequent production of cytokines and tissue factors as well as inhibition of matrix metalloproteinase activity and reduction of inflammatory cell function as a result [40]. It is well known that CRP synthesis is also IL-6 dependent, so that decrease of IL-6 plasma levels in subjects receiving statins might also be partially responsible for reduction of plasma hs-CRP concentrations, but one cannot exclude a direct impact of statins on inhibition of CRP synthesis independently from IL-6 [40]. Although TNF-α is also synthesized by adipocytes, its metabolism is not so directly associated with CRP as that of IL-6 [40]. These observations might also suggest that statins may act *via* different immune mechanisms in obesity-related chronic inflammation [40-43].

Our study has some limitations. The most important one is the relatively small study population which might not be able to show some significant relationships between compared groups. The small size of study groups makes us unable to categorize inflammatory markers into elevated and normal levels. We also cannot exclude age-related differences between compared groups; however, because of the small study population we did not consider this parameter. It should be also noted that there are very limited data obtained from other studies considering both normo- and hypertensive obese subjects and the relationship between markers of inflammation and obesity parameters, especially with newer indices such as BAI, VAI and WHtR. Therefore we have formed our conclusions very carefully. Further studies based on a larger population should be conducted to evaluate the data from our preliminary research.

## Conclusions

WHtR is a sensitive index of visceral obesity associated with chronic inflammation in obese hypertensive subjects. BAI is an obesity index correlated with hsCRP independently of hypertension and sex. High-sensitivity CRP is a more sensitive inflammatory marker associated with obesity indices than IL-6 and TNF-α. It was also noticed that lipid lowering therapy a significant impact on obesity-related chronic inflammation. These results need to be confirmed in larger studies.

## Competing interests

The authors declared that they have no competing interests.

## Authors’ contributions

MS planned the study protocol, took care about the patients and coordinate the research, took part in the data analysis and drafted the manuscript, AS took care about the patients and coordinate the research, RNW took part in the acquisition of data, MP took part in the acquisition of data, MB conceived of the study, participated in its coordination, revised it critically and prepared the final version of the manuscript, JR participated in the study coordination and took part in the data analysis. All authors read and approved the final manuscript.
